# Supplementary material for: Impaired Iron Homeostasis and Haematopoiesis Impacts Inflammation in the Ageing Process in Down Syndrome Dementia
Source: J Clin Med. 2021 Jun 29;10(13):2909. doi: 10.3390/jcm10132909 (PMC8268765; doi:10.3390/jcm10132909)
Supplement: Supplementary file 1 [file jcm-10-02909-s001.zip › jcm-1205011-supplementary.pdf]

Supplementary table S1:

Table S1. Serum analysis of control versus Down's syndrome population calculated by paired t test for Western blotting and ELISA.

**WB results**	**Paired T test**	**Two tailed**					
	Column A vs column B	Sample size: n Column A vs B	Mean (B-A) ± SEM	95% confidence interval	R^2	P value	P value summary
Aβ42	Control VS DS	A = 17 B = 17	2.000 ±1.124	-0.2885 to 4.289	0.0901	ns	
SOD1	Control VS DS	A=16 B=16	7.307 ± 0.9959	5.273 to 9.341	0.6422	<0.0001	***
S100β	Control VS DS	A=16 B=16	1.342±0.945	-0.6047 to 3.288	0.0746	0.1681	NS
Ferritin (FTL)	Control VS DS	A=20 B=20	-7.277 ± 1.570	-10.49 to -4.066	0.4257	<0.0001	****
Ferroportin (FPN)	Control VS DS	A= 16 B= 16	-1.738 ± 0.5480	-2.860 to -0.6155	0.2643	0.0037	**
Hepcidin	Control VS DS	A= 16 B= 16	1.006 ± 0.2951	0.4015 to 1.611	0.2933	0.002	**
TREM2	Control VS DS	A= 20 B= 20	-271.9 ± 84.15	-442.2 to -101.5	0.2155	0.0025	**
**Serum ELISA**	**Paired T test**	**Two tailed**					
	Column A vs column B	Sample size: n Column A vs B	Mean (B-A) ± SEM	95% confidence interval	R^2	P value	P value summary

Hepcidin	YC vs YDS	A= 23 B= 23	177.5 ± 37.02	18.27 to 171.8	0.2857	ns	
Hepcidin	OC vs ODS	A= 24 B= 24	214.5 ± 47.57	119.2 to 309.8	0.2663	<0.0001	****
Ferritin	YC vs YDS	A= 23 B= 23	-94.37 ± 12.02	-118.6 to -70.14	0.5833	<0.0001	****
Ferritin	OC vs ODS	A= 24 B= 24	-2.063 ± 18.85	-40.08 to 35.95	0.0002786	0.0465	*
IL-6	YC vs YDS	A= 23 B= 23	167.0 ± 30.57	105.3 to 228.7	0.4153	<0.0001	****
IL-6	OC vs ODS	A= 24 B= 24	324.8 ± 31.36	262.0 to 387.6	0.657	<0.0001	****
TREM2	YC vs YDS	A= 23 B= 23	-405.4 ± 125.9	-661.2 to -149.6	0.2338	0.0028	**
TREM2	OC vs ODS	A= 20 B= 20	-271.9 ± 84.15	-442.2 to -101.5	0.2155	0.0025	**

P values Statistical differences were calculated by paired t test. *p <0.01, **p <0.001, ***p <0.0001 and ****p <0.00001.
